# Selinexor, a Selective Inhibitor of Nuclear Export (SINE) compound, acts through NF-κB deactivation and combines with proteasome inhibitors to synergistically induce tumor cell death

**DOI:** 10.18632/oncotarget.12428

**Published:** 2016-10-04

**Authors:** Trinayan Kashyap, Christian Argueta, Amro Aboukameel, Thaddeus John Unger, Boris Klebanov, Ramzi M. Mohammad, Irfana Muqbil, Asfar S. Azmi, Claire Drolen, William Senapedis, Margaret Lee, Michael Kauffman, Sharon Shacham, Yosef Landesman

**Affiliations:** ^1^ Karyopharm Therapeutics Inc., Newton, MA, 02459, USA; ^2^ Department of Oncology, Wayne State University School of Medicine, Detroit, MI 48201, USA

**Keywords:** SINE, selinexor, XPO1, NF-κB, proteasome inhibitors

## Abstract

The nuclear export protein, exportin-1 (XPO1/CRM1), is overexpressed in many cancers and correlates with poor prognosis. Selinexor, a first-in-class Selective Inhibitor of Nuclear Export (SINE) compound, binds covalently to XPO1 and blocks its function. Treatment of cancer cells with selinexor results in nuclear retention of major tumor suppressor proteins and cell cycle regulators, leading to growth arrest and apoptosis. Recently, we described the selection of SINE compound resistant cells and reported elevated expression of inflammation-related genes in these cells. Here, we demonstrated that NF-κB transcriptional activity is up-regulated in cells that are naturally resistant or have acquired resistance to SINE compounds. Resistance to SINE compounds was created by knockdown of the cellular NF-κB inhibitor, IκB-α. Combination treatment of selinexor with proteasome inhibitors decreased NF-κB activity, sensitized SINE compound resistant cells and showed synergistic cytotoxicity in vitro and in vivo. Furthermore, we showed that selinexor inhibited NF-κB activity by blocking phosphorylation of the IκB-α and the NF-κB p65 subunits, protecting IκB-α from proteasome degradation and trapping IκB-α in the nucleus to suppress NF-κB activity. Therefore, combination treatment of selinexor with a proteasome inhibitor may be beneficial to patients with resistance to either single-agent.

## INTRODUCTION

Karyopherins, classified as importins and exportins, are a family of transport proteins that shuttle large (>40kDa) macromolecules between the nucleus and the cytoplasm [1, 2]. Exportin 1 (XPO1), also known as chromosomal region maintenance 1 (CRM1), is one of seven nuclear export proteins that mediate the transport of proteins, mRNA, rRNA, and snRNA out of the nucleus through the nuclear pore complex (NPC). XPO1 is the sole exporter of over 200 known cargos bearing leucine-rich nuclear export sequences (NES) [3], including major tumor suppressor proteins (TSPs) [4] such as p53, p73, p21, p27, pRb, FOXOs, BRCA1/2 and IκB-α (NFκBIA) (“NES on-line database” http://prodata.swmed.edu/LRNes/IndexFiles/names.php?sortby=nesID&orederby=DESc[5]), as well as other cell cycle regulators known to play substantial roles in cell proliferation and tumorigenesis, including survivin, NF-κB [5] and eIF4E [6]. Increased XPO1 expression is observed in many hematological and solid tumor malignancies and is correlated with poor prognosis [7–10]. Overexpression of XPO1 increases the export of TSPs to the cytoplasm, preventing them from conducting their normal cell-cycle checkpoint regulation in the nucleus [4, 11].

Using structure-based drug design, we developed highly selective small molecule inhibitors of XPO1, also known as Selective Inhibitor of Nuclear Export (SINE) compounds, which covalently bind to cysteine 528 (Cys-528) of human XPO1, located within the NES cargo binding pocket [12, 13]. XPO1 inhibition locks cargo proteins, including TSPs, in the nucleus leading to selective apoptosis of cancer cells, whereas, normal cells undergo transient cell cycle arrest [14–29]. Selinexor (KPT-330) is a first–in-class orally bioavailable SINE compound that is currently being evaluated in multiple late stage clinical trials in patients with relapsed and/or refractory hematological and solid tumor malignancies [30–32].

IκB-α (nuclear factor of kappa light polypeptide gene enhancer in B-cells inhibitor, alpha), a cargo of XPO1, inhibits the NF-κB transcription factor by sequestering it in an inactive state in the cytoplasm, thus preventing NF-κB from entering the nucleus and binding DNA [33–35]. One consequence of XPO1 overexpression in cancer cells is excessive nuclear export of IκB-α to the cytoplasm where it is inactivated by proteasome-mediated degradation [36]. The resulting up-regulation of NF-κB transcriptional activity promotes inflammation and tumorigenesis. NF-κB is activated by various intra and extra cellular stimuli including cytokines, such as TNFα [36–39]. TNFα levels in the plasma of cancer patients positively correlate with tumor growth and increased metastasis in various malignancies [40–43]. TNFα binds to the TNF-receptor and initiates signaling cascades through the IκB kinase (IKK) complex [44]. The IKK complex is composed of 3 major subunits; IKK-α (IKK1), IKK-β (IKK2) and IKK-γ (NEMO) [45]. The α- and β-subunits form the catalytically active kinase domain, whereas the γ-subunit serves a regulatory function. Activated IKK phosphorylates IκB-α leading to its dissociation from NF-κB and proteasome-mediated degradation [36, 46, 47]. Following release from IκB-α, the NF-κB complex (p65 and p50 subunits heterodimer) translocates into the nucleus and binds specific DNA sequences in the promoter region of target genes [39].

We have previously shown that selinexor inhibits NF-κB transcriptional activity even in the presence of TNFα [48]. In order to fully understand the mechanisms underlying selinexor induced NF-κB inhibition, we investigated the role of IκB-α in cancer cell response to selinexor. Our data shows that silencing IκB-α reduces the sensitivity to selinexor. We also show that NF-κB transcriptional activity is elevated in cancer cells that are naturally resistant or have acquired resistance to selinexor. Finally, we demonstrate that resistance to selinexor can be overcome by combining selinexor with proteasome inhibitors. The combination of selinexor and proteasome inhibitors excerts synergistic cytotoxicity *in vitro* and *in vivo* suggesting a promising clinical combination strategy against selinexor resistant cancers.

## RESULTS

### SINE compound resistance correlates with increased basal NF-κB activity

**Figure 1:**
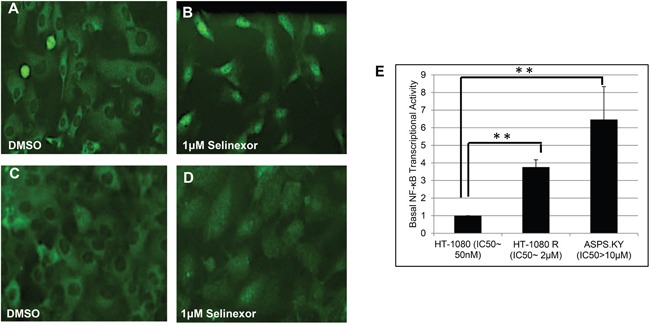
SINE compound resistant cell lines showed increased basal levels of NF-κB transcriptional activity SINE compound resistant HT-1080 (fibrosarcoma, HT-1080-R) cells were selected by continued exposure of sensitive parental cells in increasing concentrations of the SINE compound KPT-185. **A, B.** Parental and **C, D.** HT-1080-R cells were treated with 1μM of selinexor for 4 hours. SINE compound-induced nuclear retention of IκB-α was evaluated by immunofluorescence microscopy and shown to be impaired in SINE compound resistant cells. **E.** Parental-sensitive HT-1080, HT-1080-R, and ASPS-KY (Alveolar Soft Part Sarcoma) cells were tested for NF-κB transcriptional activity by ELISA assay. Equal number of cells from the 3 cell lines were lysed with RIPA buffer. The results from 2 independent assays show that higher NF-κB transcriptional activity is correlated with lower sensitivity to the cytotoxic effects of selinexor. The error bars indicate the standard deviation and the Student's t-test was used to calculate p values. **p<0.01.

We have previously described a SINE compound resistant HT-1080-R (fibrosarcoma) cell line (selinexor IC_50_: 2 μM) that was generated *in vitro* by continuous exposure of the parental cell line (HT-1080, IC_50_: 100 nM) to increasing concentrations of the SINE compound, KPT-185 [49]. Comparison of gene expression profiling between HT1080-R and parental HT-1080 cells revealed an increase of NF-κB pathway related genes in HT-1080-R cells [49]. Both cell lines showed similar levels of baseline IκB-α expression and predominantly cytoplasmic localization, however following selinexor treatment, HT-1080-R cells showed substantially less nuclear retention of IκB-α (Figure 1A–1D and [49]) compared to the parental HT-1080. To understand the role of NF-κB transcriptional activity in the context of selinexor sensitivity, we compared the basal levels of NF-κB DNA binding activity in the two cell lines. Consistent with our previous observation of increased NF-κB pathway gene expression in HT1080-R cells [49], we found ~4-fold increase in the NF-κB DNA binding activity in HT-1080-R compared to HT-1080 (Figure 1E). We then analyzed the NF-κB DNA binding activity in a naturally occurring SINE compound resistant alveolar soft part sarcoma cell line [50], ASPS-KY (IC_50_: >10 μM). We found that ASPS-KY cells had increased basal NF-κB DNA binding activity when compared to parental HT-1080 (6.5-fold) and HT-1080-R (1.7 fold), demonstrating a direct correlation between higher NF-κB activity and resistance to SINE compounds.

### IκB-α silencing decreases selinexor efficacy

**Figure 2:**
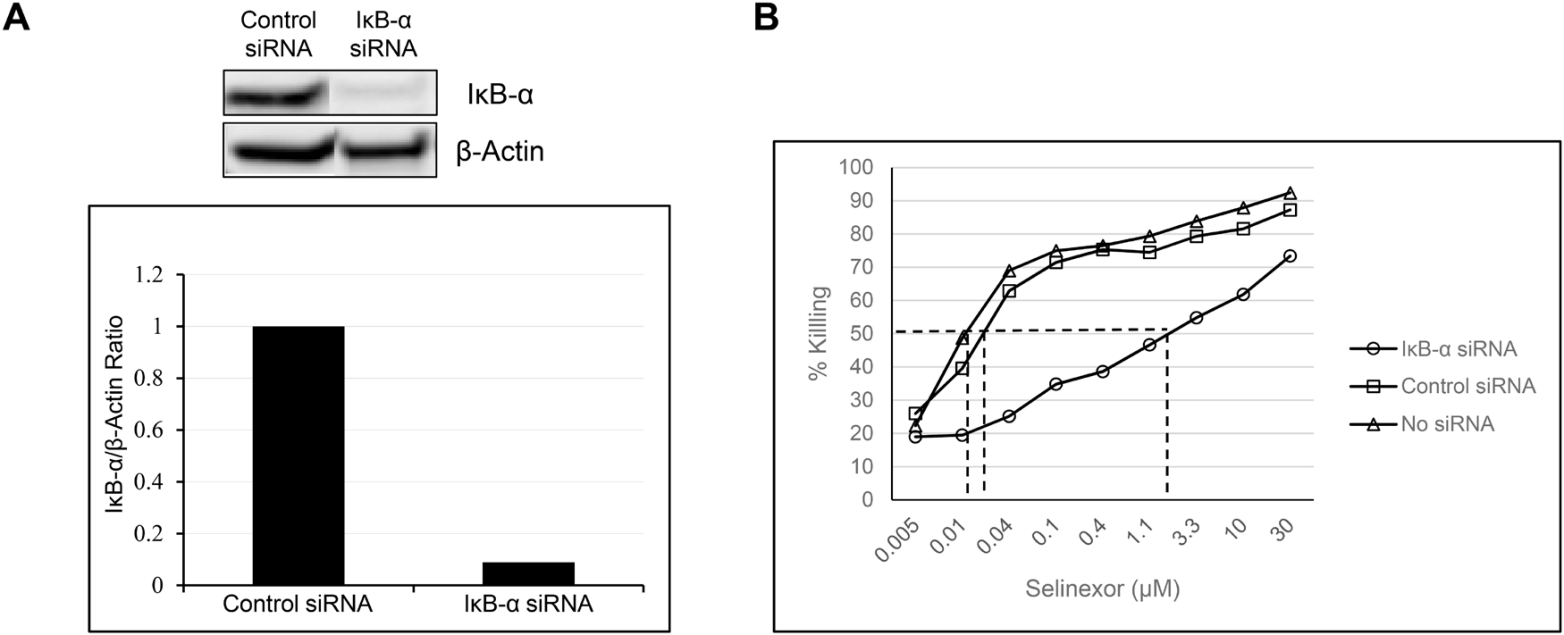
Reduction in the levels of IκB-α affects the potency of selinexor **A.** U-2 OS cells were transfected with either 40nM IκB-α or control siRNA using lipofectamine RNAiMax. At 96 hours post transfection there was a 90% reduction in the protein levels of IκB-α. **B.** The siRNA transfected and non-transfected U-2 OS cells were treated with increasing concentrations of selinexor and cells were assayed for the cytotoxic effect of selinexor 72 hours post treatment.

IκB-α inhibits NF-κB complex (p65 and p50 subunits heterodimer) nuclear translocation, DNA binding and transactivation of target genes [39]. Therefore, we hypothesized that cancer cell resistance to SINE compound-induced apoptosis may also correlate with lower expression of IκB-α. To evaluate this hypothesis, we silenced IκB-α expression in the osteosarcoma U-2 OS cell line by using siRNA and measured the cytotoxic effects of selinexor before and after IκB-α knockdown. Silencing of IκB-α with siRNA for 96 hours resulted in a 90% reduction of IκB-α protein expression (Figure 2A) and a subsequent 65-fold decrease in the cytotoxicity of selinexor (Figure 2B and Table 1). Conversely, cells transfected with control siRNA showed no change in their sensitivity to selinexor compared to the parental cell line. These results demonstrate that decreases in IκB-α protein expression contribute to the selinexor resistance in cancer cells.

**Table 1:** IκB-α knockdown in U-2 OS cells reduced selinexor cytotoxicity by 65-fold, whereas control siRNA showed no effects on selinexor potency

	Selinexor IC_50_ (μM)	Fold Change
No transfection	0.027	1
Control siRNA	0.023	0.85
IκB-α siRNA	1.85	65.5

### Combination with proteasome inhibitors overcomes selinexor resistance

**Figure 3:**
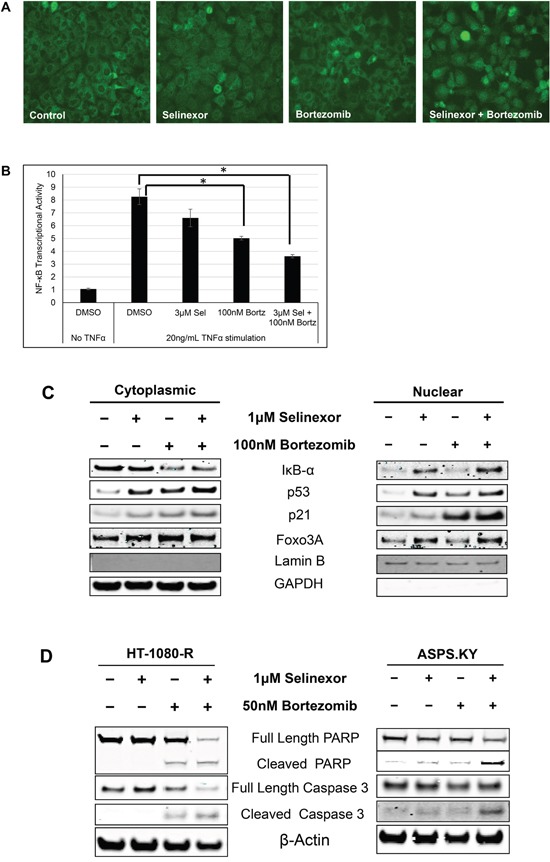
Combination with proteasome inhibitors overcomes selinexor resistance in cell lines **A.** HT-1080-R cells were treated with 1 μM of selinexor and/or 100 nM bortezomib for 12 hours. The cells were fixed and the cellular localization of IκB-α was evaluated by immunofluorescence microscopy. Selinexor treatment showed minimal nuclear entrapment of IκB-α, but combined treatment with selinexor and bortezomib further increased nuclear retention of IκB-α. **B.** HT-1080-R cells were pre-treated with 3μM of selinexor (Sel) and/or 100nM bortezomib (Bortz) for 2 hours and then exposed to 20ng/mL TNFα for 4 hours in serum free media followed by evaluation for DNA binding activity. TNFα treatment induced NF-κB transcriptional activity by 8-fold, whereas treatment with the combination of selinexor and bortezomib reduced the activity by 60% compared to 20% and 40% by the single agent respectively. The error bars indicate standard deviation and the Student's t-test was used to calculate p values. *p<0.05. **C.** Sub-cellular localization of XPO1 cargos in HT-1080-R cells treated with 1 μM of selinexor and/or 100 nM bortezomib for 12 hours was evaluated by cellular fractionation and Western blotting. The combination treatment of selinexor and bortezomib increased nuclear levels of XPO1 cargos compared to either single agent treatment. Lamin B served as a nuclear protein marker; GAPDH as a cytosolic protein marker. **D.** HT-1080-R and ASPS-KY cells were treated with 1 μM of selinexor and/or 50 nM bortezomib for 24 hours. The combination of selinexor and bortezomib was more cytotoxic than either one of the single agents as indicated by pronounced cleavage of PARP and Caspase 3 with the combination.

Proteasome inhibitors such as bortezomib and carfilzomib protect IκB-α from degradation, thereby inhibiting nuclear translocation and activation of NF-κB [51, 52]. Having demonstrated that cellular resistance to selinexor is correlated with increased NF-κB transactivation and decreased IκB-α protein expression, we sought to determine if proteasome inhibitors could overcome resistance to selinexor by preventing IκB-α degradation. Therefore, HT-1080-R cells were treated with 1 μM selinexor, 100 nM bortezomib, or a combination of both compounds for 6 hours. The cells were then fixed and stained with an IκB-α antibody and examined for sub-cellular localization. While bortezomib alone did not alter the predominantly cytoplasmic localization of IκB-α, the proteasome inhibitor enhanced selinexor-induced nuclear localization of IκB-α in HT-1080-R cells (Figure 3A). In addition, the combination treatment resulted in more inhibition of the DNA binding activity of the NF-κB (56%) compared with either drug alone (selinexor 20% and bortezomib 40%) (Figure 3B). Nuclear-cytoplasmic fractionation of HT-1080-R cells treated with selinexor, bortezomib or the combination confirmed the enhanced nuclear localization of IκB-α with the combination (Figure 3C). In addition to IκB-α, the combination also enhanced the nuclear accumulation of the tumor suppressor proteins p53, p21 and Foxo3A as compared to either selinexor or bortezomib treatment alone (Figure 3C). The enhancement of selinexor induced XPO1 inhibition by bortezomib prompted us to test the cytotoxic efficacy of the combination. Treatment of HT-1080-R and ASPS-KY cells with 1 μM selinexor or 50 nM bortezomib alone induced low levels of apoptosis (HT-1080-R by bortezomib and ASPS-KY by both single agents), whereas the combination induced marked apoptosis as demonstrated by PARP and caspase 3 cleavage (Figure 3D). In the presence of carfilzomib at its IC_25_ (4 nM), the cytotoxic potency of SINE compound, KPT-185, was enhanced by 15-fold in HT-1080-R cells (Table 2). Similarly, bortezomib enhanced KPT-185 potency by 39 folds. Cellular sensitivity to selinexor was also enhanced by 12-fold when combined with bortezomib in the selinexor- and bortezomib-resistant ASPS-KY cell line (Table 2).

**Table 2:** HT-1080-R and ASPS-KY cells were treated for 72 hours with serial dilutions of the indicated SINE compounds with/without proteasome inhibitors at concentrations below their IC_50_

	HT-1080-R	
Compound	IC_50_ (μM)	Fold Change
KPT-185	4.3	**1**
Bortezomib	0.0048	
KPT-185 + 4.1 nM Bortezomib	0.11	**39**
Carfilzomib	0.0076	
KPT-185 + 4.1 nM Carfilzomib	0.28	**15.4**
	**ASPS-KY**	
**Compound**	**IC_50_ (μM)**	**Fold Change**
Selinexor	22	**1**
Bortezomib	>>1	
Selinexor + 1μM Bortezomib	1.89	**11.64**

Proteasome inhibitors sensitized HT-1080-R and ASPS-KY cells to SINE compounds by 39- and 12-fold respectively.

### *In vivo* combination of the proteasome inhibitor, bortezomib with selinexor is more efficacious than either single agent in SINE compound resistant tumors

**Figure 4:**
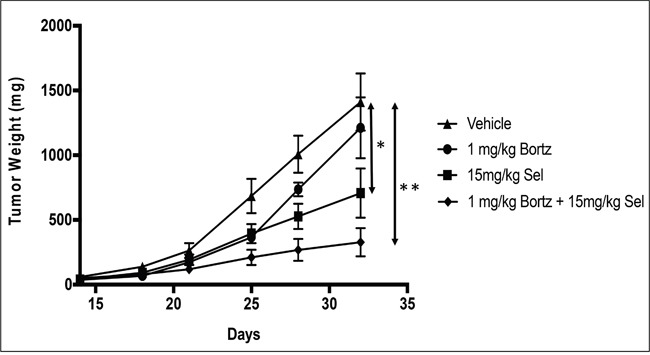
*In vivo* combination of bortezomib with selinexor is more efficacious than either single agent in the SINE compound resistant tumors HT-1080-R cells (1×10^6^) were subcutaneously injected in the flank of 3 naïve mice. Four weeks post injection, palpable tumors were formed at which time mice were euthanized, and tumors harvested and 50 mg pieces were re-implanted unilaterally into the flank of experimental groups. Bortezomib (Bortz) at 1mg/kg iv; selinexor (Sel) 15mg/kg orally or the combination of bortezomib at 1mg/kg + selinexor at 15mg/kg was administered Monday and Thursday for 2 weeks. Effects on tumor weight were recorded as described in materials and methods. Data shown are the mean for each group. The bortezomib by itself showed no significant tumor reduction. Selinexor as single agent and in combination showed 50% and 76% tumor weight reduction respectively at the end of the study (day 32). The error bars indicate SEM and the Student's t-test was used to calculate p values. *p<0.05, **p<0.01.

In order to evaluate the activity of selinexor either as a single agent or in combination with bortezomib, a sub-cutaneous xenograft model of HT-1080-R cells was established in ICR-SCID mice. The tumor-bearing mice were treated with vehicle, bortezomib (1 mg/kg; IV), selinexor (15 mg/kg; PO) or the combination of bortezomib (1 mg/kg) and selinexor (15 mg/kg) twice a week for 2 weeks. The study was terminated once tumors in the vehicle treated mice group approached 1800mg. While bortezomib treatment had little effect on tumor growth (15% inhibition) in this model, selinexor treatment resulted in 50% inhibition of tumor growth and the combination treatment resulted in 76% inhibition of tumor growth when compared to the vehicle treatment group at the end of the study (Figure 4). These results confirmed the *in vitro* finding that bortezomib treatment can sensitize SINE compound resistant tumor cells to selinexor treatment. These data suggest that combining selinexor with a proteasome inhibitor may be synergistic in cancer patients that have become selinexor or bortezomib (or other proteasome inhibitors e.g. carfilzomib or ixazomib) resistant.

### Selinexor inhibits NF-κB DNA binding and promotes nuclear localization of IκB-α and protection from degradation

**Figure 5:**
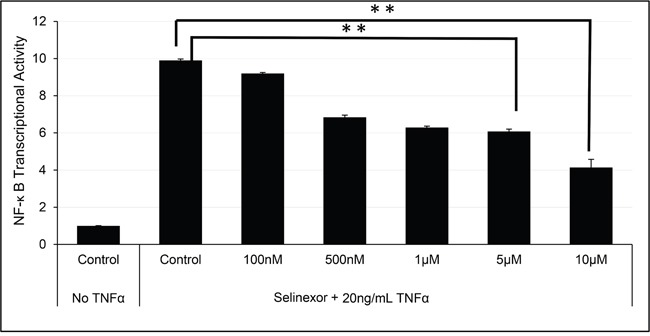
Selinexor inhibited NF-κB transcriptional activity U-2 OS cells were pre-treated with different concentrations of selinexor for 2 hours and then exposed to TNFα for 4 hours in serum free media. TNFα exposure induced NF-κB transcriptional activity by 10-fold. Selinexor inhibited NF-κB transcriptional activity in a dose dependent manner. The error bars indicate standard deviation and the Student's t-test was used to calculate p values. **p<0.01.

NF-κB is a transcriptional regulator activated by cytokines, such as TNFα [37]. Figure 5 shows a 10-fold induction of NF-κB p65 DNA binding activity upon exposure of the U-2 OS cells to TNFα. However, pre-treatment of the cells with selinexor prior to the addition of TNFα inhibits induction of NF-κB activity in a dose dependent manner with an IC_50_ of 6.72 μM. Similar results were seen in other solid [48] and hematological cell lines (data not shown).

**Figure 6:**
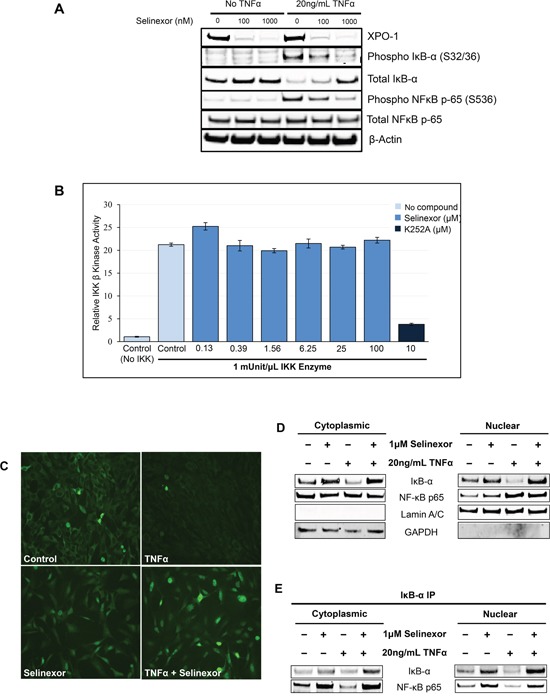
Inhibition of NF-κB transcriptional activity by selinexor is associated with nuclear localization of IκB-α and protection from degradation **A.** U-2 OS cells were stimulated with or without 20ng/mL TNFα for 2 hours before being treated with vehicle, 100nM or 1μM selinexor for the next 24 hours. Western blot of phospho-IκB-α and phosphor-NF-κB p-65 shows that selinexor reverts the pro-inflammatory effects of TNFα and selinexor also increased cellular levels of IκB-α. Selinexor induces XPO1 degradation. It is mediated by the proteasome degradation pathway (TK and YL, not shown). **B.** IKKβ kinase activity was analyzed by *in vitro* kinase assay using recombinant IKKβ, recombinant IκB-α substrate containing serine 32/36 residues and selinexor at different concentrations. IKKβ kinase activity was detected using a phosphorylation specific IκB-α antibody. Selinexor had no inhibitory effects on IKKβ kinase activity. The pan-kinase inhibitor K252A was used as a positive control for the assay. **C.** Immunofluorescence staining of IκB-α after treatment with 20ng/mL TNFα or/and 1μM selinexor for 24 hours. Selinexor induced nuclear localization of IκB-α in the presence or absence of TNFα. **D.** Cellular fractionation of U-2 OS cells shows similar increased nuclear levels of IκB-α and NF-κB p65 upon selinexor treatment even in the presence of TNFα. Lamin A/C was used as nuclear protein marker; GAPDH as a cytosolic protein marker. **E.** IκB-α immunoprecipitation (IP) and Western blotting of cytoplasmic and nuclear fractions of U-2OS cells treated with selinexor and TNFα shows that IκB-α binds to NF-κB p65 subunit both in the nucleus and cytoplasm.

In order to understand how selinexor inhibits NF-κB activation, we treated U-2 OS cells with increasing concentrations of selinexor in the presence or absence of the NF-κB activator TNFα. Then cell lysates were analyzed by Western blotting for total and phosphorylated IκB-α and the NF-κB p65 subunit protein levels. Treatment with TNFα alone induced the phosphorylation of IκB-α on serine 32/36 and the phosphorylation of NF-κB p65 on serine 536. Phosphorylation of these sites has been reported by others to be mainly IKK-dependent leading to the proteasomal degradation of total IκB-α and subsequently to the activation of NF-κB (reviewed in [44]), (Figure 6A). Conversely, treatment of U-2 OS cells with selinexor following TNFα exposure abrogated TNFα induced NF-κB and IκB-α phosphorylation, protecting IκB-α from degradation and preventing the activation of the NF-κB complex (Figure 6A and see Figure 3B). To confirm that the inhibition of IκB-α and NF-κB p65 phosphorylation by selinexor was not due to direct disruption of IKK kinase activity, increasing concentrations of selinexor were evaluated in an IKKβ kinase immunoassay using a recombinant IκB-α peptide substrate containing the target site of IKKβ phosphorylation (serine 32/36 residues). These results show that selinexor, even at concentrations as high as of 100 μM, did not directly inhibit IKKβ kinase activity (Figure 6B).

In addition to preventing IκB-α degradation, selinexor induces nuclear accumulation of IκB-α in the presence of TNFα (Figure 6C). This nuclear retention in the presence of TNFα was also confirmed by cell fractionation studies (Figure 6C). In addition to IκB-α, selinexor also induced the nuclear accumulation of NF-κB p65 subunit, a cargo of XPO1 (Figure 6D). Interestingly, immunoprecipitation of IκB-α showed that IκB-α was bound to NF-κB p65 subunit both in the nucleus and cytoplasm (Figure 6E) and that association of NF-κB p65 with IκB-α in the nucleus is enhanced in the selinexor treated cells. This data suggests that although selinexor enhances nuclear localization of the NF-κB p65 subunit, IκB-α nuclear localization is also enhanced, allowing IκB-α binding and inhibition of NF-κB activity in the nucleus, thus preventing NF-κB dependent gene transactivation.

## DISCUSSION

XPO1 is a key member of the karyopherin family of nuclear transporters and a master regulator of NES-dependent nuclear export. XPO1 overexpression is advantageous for oncogenesis and for the maintenance of the cancerous phenotype. The XPO1 mediated enhanced nuclear export of tumor suppressors leads to their exclusion from the nucleus, loss of cell cycle checkpoint regulation and therefore for aggressive uncontrolled cell growth and an anti-apoptotic phenotype [1]. Several natural and synthetic XPO1 inhibitors such as Leptomycin B, Anguinomycin A, Ratjadone A, and CBS9106 [53, 54] have been studied *in vitro* and *in vivo*. However, only selinexor has advanced to later stage clinical trials [30–32]. Selinexor covalently binds to Cys-528 in the cargo-binding pocket of human XPO1 [54] leading to trapping of XPO1 cargo proteins in the nucleus. This leads to the induction of apoptosis in cancer cells through re-activation of tumor suppressor function, while sparing normal cells [55]. In an effort to understand cancer cell resistance to SINE compounds, we established a SINE compound resistant cell line from the parental sensitive fibrosarcoma HT-1080 cell line [49]. The relatively long period of selection necessary to enrich for this resistant HT-1080 cell line (>9 months) and the fact that no mutations were identified in the cargo binding pocket of XPO1 isolated from resistant cells is very encouraging from a clinical perspective. This suggests that the XPO1 drug target is relatively resistant to the development of active-site mutations as compared to kinase targets that tend to acquire mutations and become resistant to kinase inhibitors more quickly *in vitro* [56].

Two observations in the SINE compound resistant cells that we isolated indicated that NF-κB activity is central for selinexor sensitivity. First, increased expression of inflammation-related genes is observed in the SINE compound resistant cell line HT1080-R as compared to the parental HT-1080 cells. Second, the ability of selinexor to induce nuclear localization of XPO1 cargos is reduced in SINE compound-resistant cells [49]. Of the several XPO1 cargos examined in the SINE compound-resistant cells, nuclear localization of IκB-α was most dramatically reduced. The role of NF-κB/IκB-α in selinexor sensitivity, was confirmed through measurements of high NF-κB activity in resistant cells and defining resistance of IκB-α deficient cells to selinexor (~65-fold less potent). These results suggest that functional IκB-α is required for optimal selinexor efficacy. Functionally active IκB-α inhibits NF-κB transcriptional activity by masking the nuclear localization signals (NLS) of NF-κB proteins and sequestering the NF-κB complex in the cytoplasm in an inactive form [34]. Our results further suggest that IκB-α can inhibit NF-κB activity in the nuclear compartment as well.

Proteasome inhibitors such as bortezomib and carfilzomib are known to inhibit NF-κB activity by preventing the degradation of IκB-α [51]. Since up-regulation of NF-κB activity contributes to selinexor resistance, we examined whether the combination of proteasome inhibitors with selinexor could overcome resistance to SINE compounds. Combination treatment of HT-1080-R and ASPS-KY cells with selinexor and bortezomib re-sensitized the cells to the cytotoxic effects of selinexor by 39- and 12-fold, respectively. Similar synergistic effects were observed when selinexor was combined with carfilzomib (Table 2). *In vivo* studies with HT-1080-R cells confirmed the enhanced activity of the combination observed *in vitro*. This synergy could be explained by the increased nuclear accumulation of IκB-α seen with the combination of proteasome and nuclear export inhibition. Therefore, the resulting increase in nuclear IκB-α following combination treatment translates to greater inhibition of NF-κB nuclear transcriptional activity with both XPO1 and proteasome inhibition, compared to either single mechanism alone.

Activation of the NF-κB pathway induces cell division, uncontrolled cancer cell proliferation and inflammation. Cytokines, such as TNFα, activate the NF-κB pathway by initiating phosphorylation and subsequent proteasomal degradation of IκB-α, which releases the NF-κB protein complex [39]. The NF-κB complex then translocates into the nucleus and mediates the transcription of proteins involved in cell survival, proliferation and inflammation. TNFα has been previously reported to correlate with the extent of disease in cancer patients. Significant TNFα elevation in metastatic disease when compared to localized disease has been reported in breast, prostate, pancreatic and gastric cancer patients [40–43]. Selinexor inhibits the TNFα-induced transcriptional activity of NF-κB. Our studies demonstrate that this is achieved through the inhibition of XPO1-mediated nuclear export of key members of the NF-κB pathway, namely NF-κB p65 subunit and IκB-α. Selinexor inhibits the nuclear export of both proteins, sequestering them in the cell nucleus and enabling NF-κB inactivation through IκB-α binding.

It has been previously reported that the presence of IκB-α in the nucleus prevents NF-κB from binding to DNA sequences in the promoter of target genes [57, 58]. The accumulation of IκB-α in the nucleus following exposure to selinexor could explain this inhibition of NF-κB transcriptional activity. Our immunoprecipitation studies with an IκB-α antibody show that NF-κB is bound to IκB-α both in the nucleus and in the cytoplasm. In addition, selinexor treatment increases the amount of nuclear NF-κB present in IκB-α immunoprecipitates, corresponding to an increase of the nuclear fraction of NF-κB.

Importantly, inhibition of the NF-κB signaling by selinexor is not solely achieved through the forced nuclear accumulation of IκB-α. Our data demonstrates that additional mechanisms lead to the inhibition of TNFα-induced degradation of IκB-α. Selinexor also induces inhibition of phosphorylation of IκB-α and the NF-κB p65 subunit. These sites are known to be phosphorylated by IKK. Therefore, we speculate that selinexor indirectly inhibits IKK phosphorylation of both IκB-α and NF-κB p65 subunit marking an additional way by which selinexor inhibits NF-κB activity (Figure 6A). TNFα mediated phosphorylation [44] is inhibited by selinexor in a dose dependent manner, protecting IκB-α from degradation and preventing phosphorylation and activation of the NF-κB complex. The mechanism of selinexor blockade of NF-κB-activating phosphorylation is currently a focus of active investigation in our lab.

In another report in this issue, Turner et al. examined the combination of selinexor with proteasome inhibitors in multiple myeloma cell lines and patient samples that are resistant to proteasome inhibitor treatment. The authors report that XPO1 inhibition as well as combination treatment of selinexor with bortezomib or carfilzomib sensitize the resistant cells to the cytotoxic effects of proteasome inhibitors. Together our studies highlight the NF-κB signaling as a critical pathway determining cancer cell response to selinexor and suggest a rationale for drug combination treatment to overcome drug resistance.

In conclusion, elevated NF-κB transcriptional activity may predict resistance to SINE compounds. Higher IκB-α protein expression and low NF-κB transcriptional activity are associated with SINE compound sensitivity in tumor cells. Selinexor treatment blocks the degradation of IκB-α, induces its nuclear localization and inhibits NF-κB signaling. When basal NF-κB activity is high, combination treatment of selinexor with proteasome inhibitors leads to synergistic cytotoxic activity *in vitro* and *in vivo*. Therefore, combination treatment of selinexor with proteasome inhibitors may be beneficial in patients resistant to either single agent. This work demonstrates the effect of XPO1 inhibition on the NF-κB pathway and provides a compelling rationale for combining selinexor with proteasome inhibitors as a viable option for clinical therapy.

## MATERIALS AND METHODS

### Cell culture and reagents

U-2 OS cells (#HTB-96) and HT-1080 cells (#CCL-121) were purchased from ATCC and cultured in McCoy's 5A (Gibco#12330-031) and EMEM (Corning#10-010-CV) media, respectively. Media was supplemented with 10% heat-inactivated fetal bovine serum (Corning#35-011-CV), 100 units/mL penicillin, 100 μg/mL streptomycin (Gibco#15140122) and 1Å~ GlutaMAX (Gibco#35050-061).

### Antibodies and reagents

Antibodies to Phospho IκB-α (9246), Phospho NF-κB p65 (3033), PARP (#9542), NF-κB p65 subunit (8242), Foxo3A (2497) were purchased from Cell Signaling; p21 (ab16767), IκB-α (ab32518), IKKγ (ab63255), GAPDH (ab8245) were purchased from Abcam, and p53 (sc-126), XPO1 (sc-5595), β-actin (sc-81178) and Lamin B (sc-6216) were purchased from Santa Cruz Biotechnology. Secondary antibodies were purchased from LI-COR and Invitrogen (#A11008). siRNA against IκB-α (s9512) and BLOCK-iT Alexa Fluor Fluorescent Control (#14750-100) were purchased from Life Technologies.

### Compounds

Selinexor was obtained from Karyopharm Therapeutics. Bortezomib (#S1013) and carfilzomib (#S2853) were purchased from Selleckchem. K252a (#K1639) was purchased from Sigma Aldrich.

### IκB-α silencing and cytotoxicity assays

U-2 OS cell lines were transfected with 40nM of IκB-α siRNA or 40nM BLOCK-iT using Lipofectamine RNAiMAX reagent (#13778100, Life Technologies) following the manufacturer's instructions. The transfection was performed using antibiotic free McCoy's 5A media. Twenty-four hours post-transfection, U-2 OS cells were treated with different concentrations of selinexor in triplicate for the following 72 hours. Cell viability was analyzed using CellTiter-Fluor Cell Viability Assay (#G6080, Promega) and the half maximal inhibitory concentration (IC_50_) of selinexor for each condition was calculated using XLfit. Western blotting was performed to evaluate the protein expression of IκB-α in the transfected cells.

### NF-κB transcriptional activity assay

Cells were seeded in either 6- or 12-well plates and were given overnight to adhere. Cells were pre-treated with serial dilutions of selinexor and/or bortezomib for 2 hours and then exposed to 20 ng/ml TNFα (Peprotech) for 4hrs in serum free media. After treatment, the cells were washed with PBS (Invitrogen), and lysed with RIPA buffer (Themo Scientific). The transcription activity of NF-κB in the cell lysates was measured by Chemiluminescent Transcription Factor Assay kit (Thermo Scientific Catalog# 89859) according to the manufacturer's instructions. Briefly, 1.5 mg/ml of RIPA lysed whole cell extract from each treatment were incubated in a 96-well plate bound with NF-κB biotinylated-consensus sequence. The active NF-κB transcription factor bound to the consensus sequence is incubated with NF-κB p65 primary antibody and then with a secondary HRP-conjugated antibody. A chemiluminescent substrate is added to the wells and the resulting signal is detected using a luminometer.

### Western blotting

Cells were seeded in 6-well plates at a density of 0.5×10^6^ cells/well. Post treatment, the cells were washed with 1X PBS and then lysed with RIPA buffer (#89901, Thermo Scientific) supplemented with protease inhibitors (# 05892791001, Roche) and phosphatase inhibitors (# 04906837001, Roche). The protein level of each sample was quantified and normalized using BCA assay (#23225, Thermo Scientific). 20 μg of each sample were run in 4-12% Bis-Tris Gel (Life Technologies) and later transferred to nitrocellulose membrane using iBlot Gel Transfer Kit (Life Technologies). The membranes were blocked using LI-COR blocking buffer (#927-40000, LI-COR), probed with the indicated antibodies and analyzed using Licor Odyssey.

### Cell proliferation assay

SINE compound resistant HT-1080 (HT-1080-R) and ASPS-KY cells were seeded in 96-well plates at a density of 3,000 cells per well and allowed to adhere overnight. Cells were then incubated at 37°C in a 5% humidified CO_2_ incubator for 72 hours with various concentrations of selinexor with or without bortezomib or carfilzomib at different concentrations. Cell viability was determined using CellTiter-Fluor Viability Assay (# G6082; Promega) as instructed by the manufacturer. IC_50_ was calculated using XLfit software.

### Subcellular fractionation

For nuclear/cytoplasmic protein fractionation, cells were treated with selinexor, bortezomib or both. Cells were trypsinized, washed with PBS and cellular fractionation was carried out using the NE-PER nuclear and cytoplasmic extraction kit (Thermo Scientific#78833) according to the manufacturer's instructions. Fractionation efficiency was evaluated by protein expression of subcellular marker proteins; GAPDH (cytoplasmic) and Lamin B (nuclear).

### Immunofluorescence

For the detection of IκB-α and NF-κB p65 subunit localization, cells were treated according to the study design and washed with PBS. Cells were fixed with 100% ice-cold methanol (MeOH) and permeabilized/blocked with 0.1% Tween 20, 0.3 M glycine, and 1% BSA in PBS. The rabbit secondary antibody, Alexa Fluor 488 (Invitrogen, A11008) was used for all the staining, while nuclei were stained with DAPI (Invitrogen). Protein localization was visualized with a Nikon Eclipse Ti inverted fluorescence microscope (Nikon) and monochrome camera (ANDOR) at 20X magnification.

### IKKβ kinase assay

Activity of IKKβ was quantified by IKKβ kinase assay/inhibitor screening kit (MBL #CY-1178) using recombinant IκB-α polypeptide, containing 2 serine residues, which are substrates of IKKβ. Phosphorylation of IκB-α by purified recombinant IKKβ was measured the presence of selinexor or K252A using a horseradish peroxidase conjugate of an anti- phospho IκB-α s32 specific antibody, which then catalyzes the conversion of the chromogenic substrate tetra-methylbenzidine from a colorless solution to a blue solution. The absorbance for each sample was measured using a spectrophotometric plate reader at dual wavelengths of 450/540 nm.

### Xenograft study

The efficacy of selinexor as a single agent or in combination with bortezomib was evaluated *in vivo*. 60 female ICR-SCID mice (3-4 weeks old) were purchased from Taconic farms (Germantown, NY). All IACUC approved animal experiments were carried out at Wayne State University facility. To generate xenograft tumors initially, 1×10^6^ HT-1080-R cells were injected subcutaneously in the flank of 3 naïve mice. Four weeks post initiation, palpable tumors were formed at which mice were euthanized, and tumors harvested and 50 mg pieces were re-implanted unilaterally into the flank of experimental groups. Two weeks post transplantation, tumor-bearing mice were randomized into 4 different cohorts each with 10 animals; control, bortezomib at 1mg/kg iv; selinexor 15mg/kg orally and the combination of bortezomib at 1mg/kg + selinexor at 15mg/kg. All single or combination therapy was administered Monday and Thursday for 2 weeks.
